# Retrospective Analysis of Responders and Impaired Patients with Knee Osteoarthritis Treated with Two Consecutive Injections of Very Pure Platelet-Rich Plasma (PRP)

**DOI:** 10.3390/bioengineering10080922

**Published:** 2023-08-03

**Authors:** Alain Silvestre, Pierre-Francois Lintingre, Lionel Pesquer, Philippe Meyer, Marie-Hélène Moreau-Durieux, Benjamin Dallaudiére

**Affiliations:** Clinique du Sport de Bordeaux Mérignac, 33700 Mérignac, France; alain.silvestre@me.com (A.S.); pf.lintingre@imagerie-enosis.fr (P.-F.L.); l.pesquer@imagerie-enosis.fr (L.P.); p.meyer@imagerie-enosis.fr (P.M.); mh.moreaudurieux@imagerie-enosis.fr (M.-H.M.-D.)

**Keywords:** knee joint, osteoarthritis, cartilage, intra-articular injection, platelet-rich plasma

## Abstract

Objectives: To assess the effectiveness of two consecutive intraarticular injections of PRP to treat knee osteoarthritis (KOA), discriminating between responders and impaired patients. Methods: This retrospective study included 73 consecutive patients who were referred for two intra-articular PRP injections (one week apart) for treating symptomatic moderate/severe KOA. Biological characterization of the PRP, including platelets, leukocytes and erythrocytes, was evaluated. Patient’s subjective symptoms were recorded before the treatment and 1 year after the second injection using pain VAS and WOMAC scores. Responders were defined by an improvement of 10 points on WOMAC. Results: At a 1-year follow up, we found 36 (49.3%) patients who fulfilled the criteria of responders, and 21 (28.8%) patients were impaired. A statistically and clinically significant global improvement of −29.2 ± 14.3 (*p* < 0.001) points in WOMAC score was observed 1 year after treatment in the responder group, with a higher response rate in patients with KL 2 (57.7%) compared to KL IV (28.6%). The percentage of patients with KL IV was higher in the impaired group (48.0%) compared to the responders (16.6%). As expected, the evaluation of the functionality of the knee in the impaired group indicates that it significantly worsened after one year from treatment (*p* = 0.027). However, the average pain score remained stable with no significant differences after 1 year (*p* = 0.843). No clinical complications or severe adverse events after the PRP injections were reported. Conclusion: The present study suggests that two intra-articular injections of 10 mL of very pure PRP provide pain and functional improvement in symptomatic KOA.

## 1. Introduction

Knee osteoarthritis (KOA) is a common disease associated with global knee pain and limiting flexion-related activities of daily living and exercise. It is caused by the loss of the cartilage typically in a combination of degeneration of the articular cartilage as well as abnormal biomechanical tracking of anterior, medial or, most closely, in the lateral knee parts [1,2]. Nevertheless, KOA is not limited to the cartilage and bone component, but rather affects the whole joint compartment, including the synovia and the ligaments [3]. From an epidemiology point of view, KOA prevalence is around 10% in men and 13% in women in the population over 60 years of age, which means that KOA is one of the main disability causes in old people [4]. Despite its prevalence, treatment of this painful disorder is challenging due to the variety of causes and the lack of knowledge on articular cartilage regeneration [5]. Symptomatic and conservative treatments are the main therapeutic modalities for patients with KOA. They include options such as antalgic and anti-inflammatory drugs, weight loss, physical therapy, bracing and intra-articular injections [6]. If these therapies fail, a select group of patients may require surgical intervention, such as patellofemoral replacement or complete knee arthroplasty [7,8]. A large effort has been put into finding conservative treatments able to delay natural osteoarthritis progression, with the aim of avoiding or postponing the need of important surgical interventions, such as total knee prostheses. Various clinical trials have shown that a single intra-articular injection of platelet-rich plasma (PRP) is an effective therapy for improving the clinical conditions of patients suffering from knee OA [9,10,11]. However, only a few reports have studied the effect of more than one injection of PRP [12,13,14,15,16]. In the present study, we examined retrospectively the effectiveness of two consecutive intraarticular injections of PRP to treat KOA, discriminating between responders and impaired patients.

## 2. Materials and Methods

### 2.1. Patients

The authors retrospectively reviewed 73 patients with knee OA from February 2018 to March 2020, who were aged between 20 and 85 years old and referred to our institution by sports medicine and orthopedic wards (secondary care) for intra-articular injection of PRP after failure of initial treatment with oral non-steroidal anti-inflammatory drugs and conservative physiotherapy treatment. Demographic, clinical and radiological variables such as age, sex, body mass index (BMI) and degree of radiological involvement were collected.

Inclusion criteria included patients with persistent non-traumatic KOA lasting more than 3 months, with pathologic X-ray images (at least grade II Kellgren and Lawrence), who underwent two consecutive intra-articular infiltrations of PRP and had a clinical assessment 1 year after treatment. Exclusion criteria were previous intra-articular treatment (i.e., corticoid or hyaluronic acid injections) or surgical treatment. Also, patients who received additional intra-articular treatment or surgical treatment during follow-up were excluded.

The study was registered with the “Agence Nationale de Sécurité du Médicament” (ANSM, registration number 2017-A01894-49) and in accordance with the Helsinki declaration about medical research. This retrospective study was approved by our Institutional Ethics Review Board (reference number 06-2019.2).

### 2.2. PRP Preparation and Infiltration

PRP treatments were 2 injections one week apart. The same two operators (AS or BD) specialized in musculoskeletal radiology performed PRP preparation, PRP injections and evaluations. For PRP preparation, Hy-Tissue PRP^®^ 50 (Fidia Farmaceutici SpA, Abano Terme, Italy) was employed. In brief, 45 mL of peripheral blood was collected and anticoagulated with 5 mL of 3.8% sodium citrate. The 50 mL citrated blood was centrifuged at 1800 rpm for 8 min using Duografter^®^ II (Fidia Farmaceutici SpA, Italy) and 10 mL of very pure PRP was obtained according to the manufacturer’s instructions. For quality control, 0.5 mL of PRP was used for analysis of platelets (PLT), red blood cells (RBC), and leukocytes (WBC) using an automated hematology analyzer (Stel3—Linear Chemical, Barcelona, Spain). Intra-articular injection of non-activated PRP was performed 30 min after obtention using a 21-gauge needle. Real-time ultrasound control and absence of resistance during injection were used to confirm intra-articular needle position. The patient was observed for two hours following the procedure to record any adverse event. The same procedure was repeated 1 week later. General activity involving physical exertion was forbidden for 1 month.

### 2.3. Clinical Data Assessment

Patients were examined in terms of pain and knee function. We have implemented in our routine service the systematic determination of the subjective scores VAS (Visual Analog Scale for pain) and WOMAC (Western Ontario and McMaster Universities Osteoarthritis Index for global knee function evaluation) for all patients suffering from KOA. VAS and WOMAC tests were used to assess clinical outcomes by handing the respective questionnaires to every patient. Responders were defined as patients presenting an improvement of at least 10 points in WOMAC compared to baseline. According to Ehrich et al. (2000), a level of 10 points or more of improvement or decline on WOMAC is suggested as a cut-off representing a clinically significant difference (minimal perceptible clinical improvement or MPCI) [17]. Impaired patients were defined as patients presenting an impairment of at least 1 point compared to baseline in WOMAC total score.

### 2.4. Radiographic Assessment

Prior to therapy, patients had front and lateral radiographs taken. Kellgren–Lawrence grade was assessed by the same 2 experienced radiologists (AS and BD). A consensus meeting was held in all situations where one radiologist assessed the radiograph with one rating lower or higher than the other.

### 2.5. Statistical Analysis

All statistical analyses were performed using GraphPad Prism version 7.00 for Windows, GraphPad Software, La Jolla, CA, USA. Discrete variables are described as a number and percentage and were compared between groups using the Fisher’s test. Continuous variables were described by means and standard deviations. Data symmetry was analyzed using D’Agostino and Pearson tests. Parametric tests (paired *t*-test) were used for normal distributions and the Wilcoxon matched-pairs signed-rank test (paired data) or Mann–Whitney test (unpaired data) for non-Gaussian distributions. All tests were two-sided. For all tests, *p* < 0.05 was considered significant.

## 3. Results

### 3.1. Patients

In this retrospective study, 73 patients with knee OA who received intraarticular injections of leukocyte-poor PRP (LP-PRP) from February 2018 to March 2020 in our center fulfilled all the defined inclusion/exclusion criteria. Among them, 37 were female (51%) and 36 were male (49%). The mean body mass index (BMI) was 26 ± 3.7 kg/m^2^. The mean WOMAC score at baseline was 38.3 ± 20.7. The mean pain (VAS) at baseline was 5.5 ± 2.1. Radiographic analysis revealed that 52 (71%) presented with grade II/III knee OA and 21 (29%) of patients presented with grade IV according to the classification of Kellgren and Laurence (KL).

### 3.2. Demographic Assessment: Comparison of Responders, Non-Responders and Impaired Patients

Of the total (*n* = 73), 36 (49.3%) patients fulfilled the criteria of responders, 37 (50.7%) of non-responders and 21 (28.8%) of impaired. Responders and impaired groups were similar in terms of age, sex ratio, BMI and baseline pain (Table 1). However, the WOMAC score was significatively higher at baseline in the responders compared to impaired (*p* = 0.021). The percentage of patients with KL IV was higher in the impaired group (48.0%) compared to the responders (16.6%). Considering only patients with KL II/III (*n* = 52), 30 (57.7%) patients fulfilled the criteria of responders, 22 (42.3%) of non-responders and 11 (21.2%) of impaired. Considering only patients with KL IV, (*n* = 21), 6 (28.6%) patients fulfilled the criteria of responders, 15 (71.4%) of non-responders and 10 (47.6%) of impaired.

### 3.3. Biological Characteristics of PRP Injected

Peripheral blood aspiration was standardized to 45 mL, always yielding a fixed volume of PRP (10 mL) after PPP (platelet-poor plasma) separation. PRP characterization and doses of the first and second infiltration for responder or impaired patient groups are summarized in Table 2.

There were no significant differences in the PLT, RBC or WBC doses administered in each PRP injection. The preparation process leads to the obtention of a PRP with a high platelet composition (>95%) and a very good reproducibility regarding platelet purity (coefficient of variation <3%). The PRP obtained using this method is classified as ABA (leucocyte-poor (LP-PRP) based on the DEPA classification system [18].

### 3.4. Clinical Assessment

**Figure 1:**
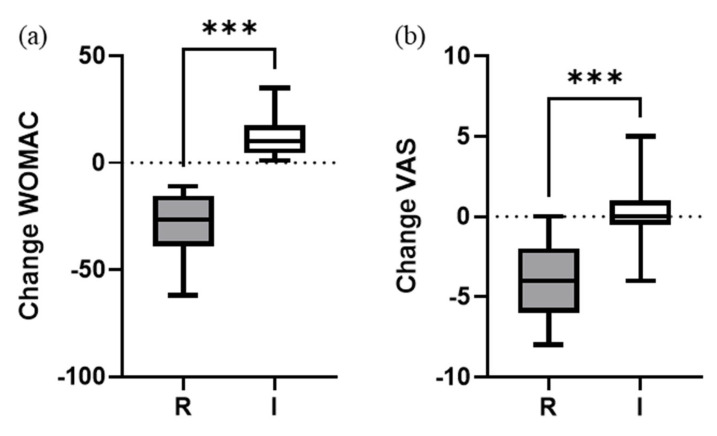
Change of (**a**) WOMAC and (**b**) VAS pain scores 1 year after PRP treatment. Absolute variation from baseline. Box and whisker plots represent the median, the lower and upper quartile, and the min and max, respectively. Mann–Whitney test (two-tailed) was used to compare groups. *** (*p* < 0.001). R = responders; I = impaired.

A statistically significant overall global improvement of −11.8 ± 21.3 (*p* < 0.001) points in WOMAC score was observed 1 year after treatment. This significative improvement was also observed in VAS pain change (−2.3 ± 2.6; *p* < 0.001) (Figure 1).

The percentage of responder patients was similar to non-responders (49% vs. 51%, respectively). When comparing responder vs. impaired (Table 3), we found significant change improvements in WOMAC and VAS scores for responders (*p* < 0.001). As expected, the evaluation of the functionality of the knee in the impaired group showed that it significantly worsened one year after the treatment (*p* = 0.027). However, the average pain score remained stable with no significant differences after 1 year (*p* = 0.843). No clinical complications or severe adverse events after the PRP injections were reported.

## 4. Discussion

This study allowed an overall assessment of the efficacy of two consecutive intraarticular infiltrations of PRP in our routine care. In brief, the administration of two intraarticular injections of a high volume of autologous pure PRP with an average platelet dose provided significant clinical benefits (responders) to 49.3% of patients with KOA with grade II to IV one year after treatment. This result could be attributed to the inclusion of patients with severe KOA. In fact, the proportion of responder patients with KOA was 57.6% and 28.6% for KL II/III and IV, respectively. Other authors also reported that severe forms of KOA respond less well to PRP treatments. Saita et al. (2021) [19] performed a retrospective study with a higher number of patients (517), assessing the effectiveness of three injections of PRP (LP-PRP; 4–5 mL, average PLT concentration = 475.4 × 106/mL) for treating KOA grades II/III/IV. The responder rate according to OMERACT-OARSI responder criteria at 12 months was 75.2%, 66.35, and 50.9% for patients with KOA grade II, III and IV, respectively, demonstrating that the severity of KOA was a predictor of poor PRP therapy outcomes. A different result was obtained by Chopin et al. (2023) [20] who studied the clinical benefits at 7 months after two consecutive intraarticular injections of PRP (3–6 mL, LP-PRP) on 197 patients with moderate–severe KOA (KL II/III/IV), showing no statistical differences between the number of responders vs. non responders in the function of the severity of the KOA.

A similar study was conducted by Bec et al. (2021) [21] who also targeted KOA grades II/III/IV but only found a responder rate (based on an increase in KOOS change in 10 points) of 45% at 6 months (34 responders/75 patients). This study differs from the current one on several points: (a) the final follow up was shorter (6 months), (b) the mean volume of pure PRP injected, dose of platelets and the number of interventions was lower compared to our protocol (6.8 mL PRP; 2.6 × 109 platelet dose, one single injection, respectively), and (c) the responders definition was not only based on WOMAC score but using the OMERACT OARSI criteria (Outcome Measures in Rheumatology, Osteoarthritis Research Society International) [22], which also considers pain and patients global assessment. Another similar study was conducted by Guillibert et al. [23] where the responder rate reached 80% based on a responder rate definition based on the KOOS score at a 6-month follow up. This study employed a very pure PRP volume injection more similar to our study (8.8 mL) but lower PLT dose (2.6 × 109) and only a single injection. Additionally, the study included only patients with mild to moderate KOA (KL II/III), excluding those with severe KO (KL IV). In our series, we employed a higher volume of very pure PRP in each knee injection (10 mL) based on the articular capacity of the knee to favor a better distribution of PRP throughout the joint [24,25]. We also consider that the inclusion of patients with KOA KL IV is important because it gives a broader idea about the effectiveness of the treatment in the early or late stages of osteoarthritis. The concept of very pure PRP was suggested by Magalon et al. [18], where it was considered that PRP is very pure when the relative composition of platelets is greater than 90%. The characterization of the PRP in our study showed that the very pure PRP consisted of a high volume of plasma with a high quantity of platelets and very low WBC and RBC content; the latter is known for being potentially harmful when injected into a knee [26,27,28]. It is remarkable that the dose of PLT used in our study was on average two times higher than the dose employed by Guillibert et al. and Beck et al. Even though up to now it has not been clearly demonstrated that platelet dose correlates with clinical effectiveness [29,30,31,32], we believe that this factor could be additionally relevant for obtaining a high rate of responders with a high level of functional improvement of the knee at 1 year after treatment (mean WOMAC change of 67%). However, Bansal et al. (2021) [33] employed a higher PLT dose (10 × 109; 10 mL PRP; one single intraarticular injection) for treating patients with KOA KL II/III, showing a small improvement in function and pain scores at a 1-year follow up (WOMAC: baseline: 55.0 (52–56); 1 year: 51.9 ± 7.4; IKDC: baseline: 53.6 ± 6.34; 1 year: 62.8 ± 6.24). It seems that one of the factors that could be more relevant for influencing the magnitude of clinical efficacy is the number of PRP infiltrations rather than the PLT dose. It has been reported in a recent meta-analysis of randomized controlled trials (RCTs) that multiple PRP cycles seems to be more effective than a single injection [34]. A different strategy that could have been used to improve the number of responding patients in severe KOA consists of a combined protocol of intraosseous and intraarticular PRP infiltrations [35,36]. This technique has shown a greater number of responders compared to the intra-articular injection technique [37]. As a retrospective study, the absence of a placebo or control group is an important limitation. Even though the patients included in this study suffered from chronic knee pain not responding to other known conservative therapies for more than a year, the effectiveness of the PRP therapy itself could have been attributed, at least in part, to the placebo effect. Nevertheless, a retrospective study can be considered acceptable for investigating the determinants of PRP therapy effectiveness, as well as for determining to what extent its effectiveness depends on KOA severity. The absence of a periodic follow up until 1 year without intermediate assessments could also be a source of bias. We have not included data on the correlation of platelet dose with clinical efficacy because (a) the platelet dose interval was quite narrow (Table 2) and (b) in our opinion, it is not possible to calculate a correlation between the change in VAS or WOMAC at a specific follow up time to a PLT dose since we had two consecutive interventions with two different PLT doses. Despite these limitations, the study provided further insights towards a better understanding of the biological activity of a high volume, very pure PRP injection for treating KOA.

## 5. Conclusions

The present study suggests that two intra-articular injections at a high volume of very pure PRP provides pain and functional improvements in moderate symptomatic KOA in 49% of the patients after 1 year of the treatment, apparently with better results in patients with grade II osteoarthritis, with a low percentage of impaired patients (29%). More studies will be required to confirm our findings and to elucidate the reasons why there are patients who do not respond or even worsen after receiving PRP treatment.

## Figures and Tables

**Table 1:** Baseline clinical characteristics and demographics of responders and impaired patients.

	PRP Responders (*N* = 36)	PRP Impaired (*N* = 21)	*p*-Value
Gender (M:F), n (%)	17:19 (47:53)	11:10 (52:48)	0.787
Age (years), mean/SD	52.6 ± 14.0	53.9 ± 13.9	0.821
BMI (Kg/m^2^), mean/SD	25.6 ± 4.0	26.0 ± 2.8	0.541
KL II-III, n (%)	30 (83.0)	11 (52.0)	0.025
KL IV, n (%)	6 (16.6)	10 (48.0)	
WOMAC baseline, mean/SD	43.8 ± 19.65	32.5 ± 20.1	0.021
VAS baseline, mean/SD	5.8 ± 2.1	4.8 ± 2.2	0.076

BMI, body mass index; VAS: visual analog scale. WOMAC: Western Ontario and McMaster Universities Osteoarthritis Index. Data are provided as mean ± SD (standard deviation), percentage or absolute values.

**Table 2:** Biological characteristics of PRP injected (*N* = 73) responders and impaired patients.

	PRP Responders		PRP Impaired		
	1st PRP	2nd PRP	1st PRP	2nd PRP	
	Mean ± SD	Mean ± SD	Mean ± SD	Mean ± SD	*p* Value
Injected volume (mL)	10.0 ± 0.0	10.0 ± 0.0	10.0 ± 0.0	10.0 ± 0.0	-
Platelet concentration (10^6^/mL)	573 ± 134	559 ± 139	573 ± 134	573 ± 134	-
Leukocyte concentration (10^6^/mL)	0.5 ± 0.2	0.5 ± 0.2	0.5 ± 0.2	0.5 ± 0.2	-
Red blood cells concentration (10^9^/mL)	0.01 ± 0.00	0.01 ± 0.00	0.01 ± 0.01	0.01 ± 0.00	-
Quantity of injected platelets (10^6^)	5724 ± 1344	5589 ± 1392	5553 ± 1363	5778 ± 1271	0.928
Quantity of injected leucocytes (10^6^)	4.5 ± 2.2	4.8 ± 2.3	4.0 ± 3.3	6.0 ± 8.6	0.499
Quantity of injected red blood cells (10^9^)	0.1 ± 0.0	0.1 ± 0.0	0.1 ± 0.0	0.1 ± 0.0	0.467
Relative composition					
Platelets (%)	96.7 ± 1.6	96.1 ± 2.7	96.9 ± 1.6	96.5 ± 1.3	-
Leukocytes (%)	0.1 ± 0.0	0.1 ± 0.0	0.1 ± 0.1	0.1 ± 0.1	-
Red blood cells (%)	3.3 ± 1.6	3.8 ± 2.7	3.0 ± 1.6	3.4 ± 1.2	-

Data are provided as mean ± SD (standard deviation). One-way ANOVA was employed for determining differences between groups.

**Table 3:** Means at baseline and at 1-year follow up and significance of intergroup changes measured as VAS pain and WOMAC score. VAS: visual analog scale. WOMAC: Western Ontario and McMaster Universities Osteoarthritis Index. Data are provided as mean ± SD (range). CI: confidence interval; 95% CI referred to change.

	Groups			
Variable	Responders	*p*-Value	Impaired	*p*-Value
WOMAC (global)				
Baseline	43.8 ± 19.6	<0.001	32.5 ± 20.1	0.027
1 year	14.6 ± 12.7		44.7 ± 20.0	
Change	−29.2 ± 14.3		−12.1 ± 9.1	
95% CI	−34.1, −24.3		7.9, 16.4	
VAS				
Baseline	5.8 ± 2.1	<0.001	4.8 ± 2.2	0.843
1 year	1.9 ± 1.4		4.9 ± 1.7	
Change	−3.9 ± 2.2		0.1 ± 1.9	
95% CI	−4.6, −3.1		−0.7, 1.0	

## Data Availability

Data are available upon reasonable request addressed to the corresponding author.

## Abbreviations

PRP	Platelet-rich plasma
VAS	Visual Analogue Scale
WOMAC	Western Ontario, McMaster Universities Osteoarthritis Index
KOA	Knee osteoarthritis
MPCI	Minimal perceptible clinical improvement
